# TACE combined Lenvatinib plus Camrelizumab versus TACE alone in efficacy and safety for unresectable hepatocellular carcinoma: a propensity score-matching study

**DOI:** 10.1186/s12885-024-12484-3

**Published:** 2024-06-11

**Authors:** Zhihong Tang, Tao Bai, Tao Wei, Xiaobo Wang, Jie Chen, Jiazhou Ye, Shangqi Li, Meng Wei, Xingzhi Li, Youzhi Lin, Juan Tang, Lequn Li, Feixiang Wu

**Affiliations:** 1 Department of Hepatobiliarypancreatic-Splenic Surgery, Guangxi Medical University Cancer Hospital, Nanning, China; 2 Key Laboratory of High-Incidence Cancer Prevention & Treatment, Ministry of Education, Nanning, China; 3 Hepatobiliary Surgery Department, The Fourth Affiliated Hospital of Guangxi Medical University, Liuzhou, China

**Keywords:** Unresectable hepatocellular carcinoma, Camrelizumab, Transcatheter arterial chemoembolization, Lenvatinib

## Abstract

**Backgrounds:**

To compare the efficacy and safety of transcatheter arterial chemoembolization (TACE) combined Lenvatinib plus Camrelizumab (TLC) in unresectable hepatocellular carcinoma (uHCC) with those of TACE alone .

**Methods:**

A retrospective analysis was performed on 222 patients with uHCC who were treated between September 2013 and Jun 2023. One group received TACE + lenvatinib + camrelizumab (TLC) (*n* = 97) and another group received TACE alone (*n* = 151). Efficacy and safety were compared after propensity score matching between the TLC and TACE groups.

**Results:**

After propensity matching, the TLC group had higher objective response rate (ORR) (88.6% vs. 28.6%, *P* < 0.001), disease control rate (DCR) (94.3%% vs. 72.9%, *P* < 0.001), and conversion rates before and after propensity matching were 44.1% and 41.4%, respectively, compared with the TACE group. The median progression free survival (PFS) was longer in the TLC group than in the TACE group (12.7 vs. 6.1 months, *P* = 0.005). The median overall survival (OS) was longer in the TLC group than in the TACE group (19.4 vs. 13.0 months, *P* = 0.023). Cox multivariate analysis with different modes of adjustment showed that treatment was an independent influencing factor of PFS and OS. The interaction analysis showed that cirrhosis and Child-Pugh stage an interactive role in the PFS of different treatment. Decreased AFP after treatment portends higher ORR and DCR.

**Conclusion:**

TACE combined Lenvatinib plus Camrelizumab regimen was safe and superior to TACE alone in improving PFS, OS, and tumor response rates for unresectable recurrent HCC patients.

**Supplementary Information:**

The online version contains supplementary material available at 10.1186/s12885-024-12484-3.

## Introduction

Primary hepatocellular carcinoma (PLC) is the third leading cause of cancer-related death. It is also one of the most common malignancies in the world, with China accounting for 45.27% of cases and 47.12% of deaths [1]. According to the. Hepatocellular carcinoma (HCC) is the most common histologic type of PLC, accounting for 75–85% of all cases, and Hepatitis B virus (HBV) infection is a significant cause of HCC in China. Most HCC is detected at the middle or advanced stage, making surgical resection unfeasible [2]. The 5-year survival rate is less than 20%, and the prognosis is poor [3].

Transcatheter arterial chemoembolization (TACE) is the basic local treatment for HCC stage B in Barcelona clinical liver cancer (BCLC) and also a recommended treatment for advanced HCC. According to BCLC strategy for prognosis prediction and treatment recommendation (the 2022 update) [4], TACE is recommended as the primary treatment for BCLC-B stage HCC that exceeds the Milan criteria. Systemic therapy is recommended only for BCLC-B patients who are not candidates for TACE for any reason [5, 6]. In BCLC-C stage TACE has also been suggested to be as effective as sorafenib in patients with liver-only involvemen [7, 8]. No positive trial results of TACE combined with systemic therapy have been obtained, so evidence-based recommendations cannot be made [9–11]. According to AASLD Practice Guidance of hepatocellular carcinoma [12], TACE is the primary treatment option for patients with BCLC Stage B HCC [13, 14]. Several trials comparing TACE alone versus TACE with multikinase inhibitors (mTKIs) failed to show significant improvements in progression free survival (PFS) or overall survival (OS) [15–17]. Based on current data, the AASLD advises against combination therapy outside of clinical trials. However, TACE alone can not effectively control tumor progression and prolong patient survival in advanced HCC. The objective response rate (ORR) of TACE alone was only 40.3% ~ 52.0%, median progression free survival (PFS) was 3.6 ~ 13.5 months and OS was 10.8 ~ 19.9 months [18, 19]. In addition, the efficacy of TACE will decrease with the increase of treatment times [20] Repeated TACE treatment will aggravate liver damage. For the treatment of advanced HCC, the rate of conversion after TACE was low, approximately 9.8% [21].

2022 Chinese clinical guidelines on the management of hepatocellular carcinoma proposed that multi-modality therapy, such as adding immunotherapy-based systemic therapy to local therapy, should be more actively applied to advanced HCC to improve the surgical conversion rate [22].The actual clinical treatment that combines local treatment with systemic treatment are widely accepted in China because of the high tumor response rate and conversion to resection rate, with controlled toxicity [23]. Because of the heterogeneity of local treatments and systemic treatments, the current study focused on the triple therapy of TACE combined with tyrosine kinase inhibitor (TKI) plus anti–PD-1 antibodies.Many real world studies and retrospective cohort study have confirmed that the triple therapy with Chinese characteristics is an effective conversion therapy regimen with a significant objective response rate (ORR), conversion potential, and satisfactory safety profile [24–29]. Unfortunatel, the current studies are mainly observational real-world studies and series of case reports. None of such study to compared TACE combining lenvatinib (TKI) and camrelizumab (anti–PD-1 antibodies) with TACE alone by now. In order to address this vital knowledge gap, we conducted a retrospective study.

## Methods

### Study design and patients

The clinicopathological data of patients with uHCC treated in Guangxi Medical University Cancer Hospital from September 2013 to June 2023 were collected retrospectively. Inclusion criteria includes: 18–80 years old; Patients with histopathologically or clinically proven hepatocellular carcinoma who are not candidates for resection.; Liver function Child-Pugh grade A or B (≤ 7 points); Eastern Cooperative Oncology Group performance status (ECOG PS) score 0–1; At least one measurable lesion; It has good bone marrow and organ function. Exclusion criteria: Patients with recurrent HCC; Any contraindications to TACE, lenvatinib and camrelizumab; Combined with other malignant tumors; Received local treatment or systemic treatment other than lenvatinib or camrelizumab before and after TACE treatment; Gastrointestinal bleeding occurred in the past 6 months; Uncontrolled pleural effusion, pericardial effusion, or moderate or above ascites; The patient had active infection. Patients who met the above inclusion and exclusion criteria were divided into TACE group and TACE + lenvatinib + camrelizumab (TLC) group according to the treatment method.

Ethics committee approval was obtained from Guangxi Medical University Cancer Hospital (LW20211105). The informed consent of this study was obtained from all subjects and/or their legal guardian(s).

### Treatment procedure

TACE was performed by experts with rich surgical experience. TACE includes traditional TACE (C-TACE) and drug eluting beads TACE (d-tace). Using the Seldinger technique, a 4 F to 5 F Fallot catheter was delivered to the abdominal aorta from the superficial femoral vein via fluoroscopy, and a catheter was delivered to the abdominal cavity after local anesthesia. Vascular anatomy of the feeding arteries and tumor periphery was performed by introducing 4 F to 5 F catheters into the microcatheter (Callispheres®, Heng Rui Callisyn Biomedical, 20,183,770,117) into the tumor arteries. For patients conducting c-TACE, 5–15 mL lipiodol (Andre Guerbet, Aulnay-sous-Bois, France) is mixed with chemotherapy drugs as drug carrier, and different diameter microsphere (8spheres®, Heng Rui Callisyn Biomedical, 20,153,131,072) are injected into tumors as embolic agents. Patients receiving D-TACE were embolized with chemotherapeutic drugs loaded with drug-eluting beads (Callispheres®, Heng Rui Callisyn Biomedical, 20,153,131,072). The treatment is deemed to be completed until the blood flow almost stops. TACE was followed up and evaluated every 4–8 weeks. TACE was performed as needed when the lesion had no complete necrosis and an active lesion area greater than 50%of the baseline.

TKI and PD-1 inhibitor are administered from one week after TACE until the patient had progressed, unacceptable toxicity, death or discontinuation for any reason. Camrelizumab® (Jiangsu Hengrui Pharmaceuticals, S20180016) 200 mg intravenousn drip once every 3 weeks and Lentivanib (LENVIMA®, Merck Sharp & Dohme, H20180052) 8 mg orally once daily. Patients were followed up for the first time 4 to 8 weeks after treatment. Efficacy assessments were performed every 2 ~ 4 months. The deadline is Jun 8, 2023. Once the patient had met the criteria for resectable HCC, hepatic resection was performed after obtaining the patient’s consent. (Child-Pugh score < 7; ECOG PS ≤ 1; No extrahepatic lesions; The vascular structure of the liver is intact and the remaining liver volume is sufficient) [18].

### Definitions

Imaging examinations (positron emission tomography-computed tomography, magnetic resonance imaging and enhanced computed tomography) were performed before and after treatment and evaluated by two independent physicians. Tumor response was defined as the best response at all time points and was assessed according to the modified Response Evaluation Criteria in Solid Tumors (mRECIST) The primary endpoint was PFS (from treatment to disease progression or death from any cause). The secondary end points were OS (from treatment to death from any cause), ORR, DCR, and surgical conversion rate. Safety assessment adverse reactions (AES) were collected and evaluated according to the common terminology standard for adverse events (CTCAE) 5.0.

### Statistical analyses

This study was analyzed by SPSS 24.0 statistical software (IBM, Armonk, NY, USA) and EmpowerStats (https://www.empowerstats.net/en//). The normal distribution of continuous variables is expressed as median (Q1, Q3) or mean ± standard deviation, and the non-normal distribution is expressed as quartile, which is compared by t-test or Mann Whitney test. The χ^2^ test or Fisher exact test was used, and counting variables were expressed as the number and percentage of cases used. Survival curves were plotted using the Kaplan Meier method, and log-rank tests were used to compare survival curves. Univariate analysis was performed using Cox risk models to identify independently associated factors for PFS and OS. We performed a stratified analysis to determine whether treatment effects differed across subgroups by age, sex, stage of BCLC, target tumor size, AFP, HBV-related, ECOG PS, tumor number, large vessel invasion, Child-Pugh grade, type of TACE, and cirrhosis. Multiple interactions were estimated by adding terms to the interaction list. For each end point, two multivariate models were constructed based on treatment selection. The second quartile or lower quadrant was used as a reference group. In model 1, the covariates were adjusted for large vascular invasion, stage of BCLC and AFP; in model 2, we further adjusted for stage of BCLC, target tumor size, AFP, ECOG PS, tumor number, large vessel invasion, Child-Pugh grade and type of TACE. To adjust for treatment-distribution imbalances, we performed a propensity score matching (PSM) analysis. The TACE group and TLC group were matched in a 1:1 ratio to maximize the propensity score to 0.05 caliper value. Propensity matching was performed according to age, BCLC stage, tumor number, large vascular invasion, Child-Pugh grade and cirrhosis. *P* < 0.05 was considered to indicate a statistically significant difference.

## Results

### Patients baseline characteristics

**Fig. 1:**
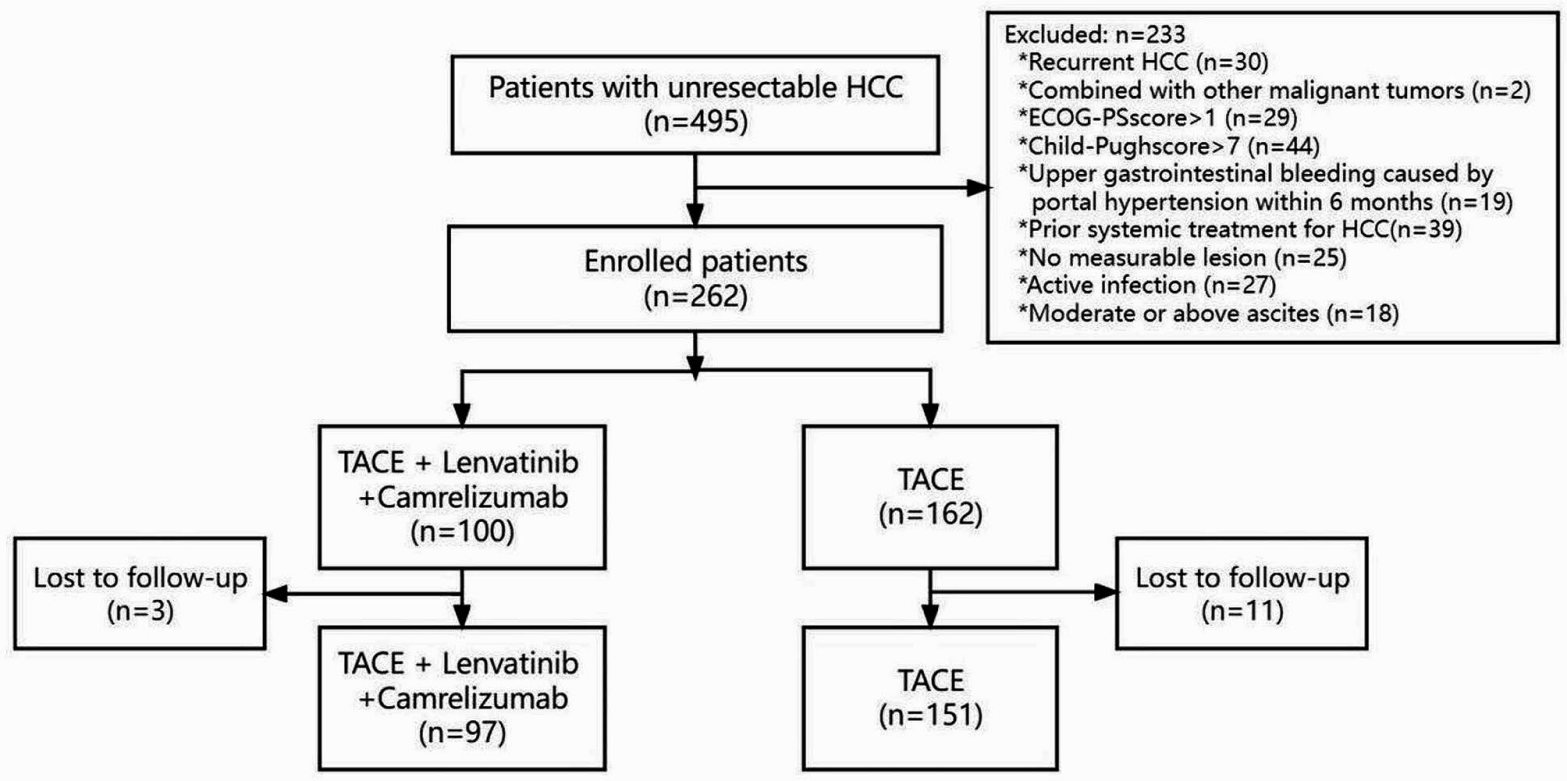
Schematic diagram of the patient selection process. HCC, hepatocellular carcinoma; ECOG-PS, Eastern Cooperative Oncology Group performance status; BCLC, Barcelona Clinic Liver Cancer; TACE, transcatheter arterial chemoembolization

From September 2013 to June 2023, 262 uHCC patients were enrolled, with 233 excluded (Fig. 1). The baseline characteristics of demographic and clinical variables in TLC group (*n* = 97) and TACE group (*n* = 151) are described in Table 1. As of Jun 8 2023, 43 patients in the TLC group had progressive disease, 25 patients had died, and the median follow-up was 11.2 (1.1–34.20) months; 130 patients in the TACE group had progressive disease, 115 patients had died, and the median follow-up was 10.60 (1.40–65.70) months. There were differences in baseline characteristics between the TLC and TACE groups in type of TACE, BCLC stage, large vascular invasion, Child-Pugh grade and cirrhosis. After propensity score matching, a total of 70 pairs of paired patients were enrolled, and the baseline characteristics between the groups were balanced.

**Table 1:** Baseline demographic and clinical characteristics of patients before and after PSM

Variable	Before PSM				After PSM		
	TLC(*n* = 97)	TACE(*N* = 151)	*P* value		TLC(*n* = 70)	TACE(*N* = 70)	*P* value
Age (year)	49.0 (42.0–57.0)	54.0 (45.50–61.0)	0.027		50.0 (45.0–57.0)	50.00 (45.00–58.00)	0.835
Gender			0.463				0.464
Female	7 (7.2%)	15 (9.9%)			3 (4.2%)	6 (3.6%)	
Male	90 (92.8%)	136 (90.1%)			67 (95.7%)	64 (91.4%)	
Type of TACE			< 0.001				0.309
C-TACE	37 (38.1%)	101 (66.9%)			29 (41.4%)	35(50.0%)	
D-TACE	60 (61.9%)	50 (33.11%)			41 (58.6%)	35 (50.0%)	
BCLC stage			< 0.001				0.900
A	16 (16.4%)	34 (22.5%)			13 (18.5%)	15 (21.5%)	
B	27 (27.8%)	75 (49.7%)			27 (38.6%)	27 (38.5%)	
C	54 (55.8%)	42 (27.8%)			30 (42.9%)	28 (40.0%)	
Target tumor size (cm)	9.99 (7.8–12.5)	9.60 (7.00-13.66)	0.591		9.54 (7.58–12.17)	9.74 (7.25–13.95)	0.820
AFP (ng/mL)			0.273				0.612
>400	49 (50.5%)	64 (42.4%)			35 (50.0%)	38 (54.3%)	
≤ 400	48 (49.5%)	87 (57.6%)			35 (50.0%)	32 (45.7%)	
HBV-relatived			0.245				0.385
Yes	85 (87.6%)	124 (82.1%)			55 (78.6%)	55 (78.6%)	
No	12 (12.4%)	27 (17.9%)			11 (15.7%)	15 (21.4%)	
ECOG PS			0.739				0.853
0	70 (72.2%)	106 (70.2%)			49 (70%)	50 (71.4%)	
1	27 (27.8%)	45 (29.8%)			21 (30.0%)	20 (28.6%)	
Cirrhosis			0.009				0.329
Yes	79 (81.4%)	100 (66.2%)			55 (78.6%)	50 (71.4%)	
No	18 (18.6%)	51 (33.8%)			15 (21.4%)	20 (28.6%)	
Tumor number			< 0.001				0.254
1	48 (49.5%)	45 (29.8%)			29 (41.4%)	27 (38.6%)	
2	17 (17.5%)	12 (8.0%)			14 (20.0%)	8 (11.4%)	
≥ 3	32 (33.0%)	94 (62.3%)			27 (38.6%)	35 (50.0%)	
Large vascular invasion			< 0.001				0.863
Yes	51 (52.6%)	110 (72.9%)			29 (41.4%)	28 (40.0%)	
No	46 (47.4%)	41 (27.2%)			41 (58.6%)	42 (60.0%)	
TBIL (umol/L)	15.30 (10.50–20.80)	15.90 (11.25–20.85)	0.337		14.30 (10.30-19.15)	18.05 (12.93–21.95)	0.064
ALB(g/L)	37.50 (34.40–39.50)	36.10 (33.10-39.25)	0.091		37.50 (33.55–39.60)	37.05 (33.12–40.42)	0.487
PT (sec)	12.50 (11.60–13.50)	12.60 (11.60–13.60)	0.597		12.65 (11.09–13.67)	12.55 (11.60–13.20)	0.470
ALT(U/L)	42.00 (31.00-66.60)	44.00 (35.00–64.00)	0.837		39.50 (30.25–62.50)	47.00 (33.50–60.00)	0.901
AST(U/L)	62.00 (45.00–89.00)	68.00 (45.50–90.00)	0.414		61.50 (47.00-88.50)	65.5 (47.00-87.75)	0.365
Cr(mmol/L)	77.00 (66.00–85.00)	77.00 (66.00-86.50)	0.228		77.00 (67.25–84.75)	76.00 (66.00–87.00)	0.286
Child-Pugh grade			0.005				0.900
A	88 (90.7%)	116 (76.8%)			62 (88.6%)	59 (84.3%)	
B	9 (9.3%)	35 (23.2%)			8 (11.4%)	11 (15.7%)	
ALBI grade			0.986				0.930
1	24 (24.7%)	36 (23.8%)			20 (28.6%)	18 (25.7%)	
2	71 (73.2%)	112 (74.2%)			49 (70.0%)	51 (72.9%)	
3	2 (2.0%)	3 (2.0%)			1(1.4%)	1 (1.4%)	

BCLC: Barcelona Clinic Liver Cancer; ECOG PS: Eastern Cooperative Oncology Group performance status; TBIL: Total bilirubin; ALB: albumin; PT: Prothrombin time; AFP:α-fetoprotein; Hepatitis B virus

### Tumor response evaluation

**Fig. 2:**
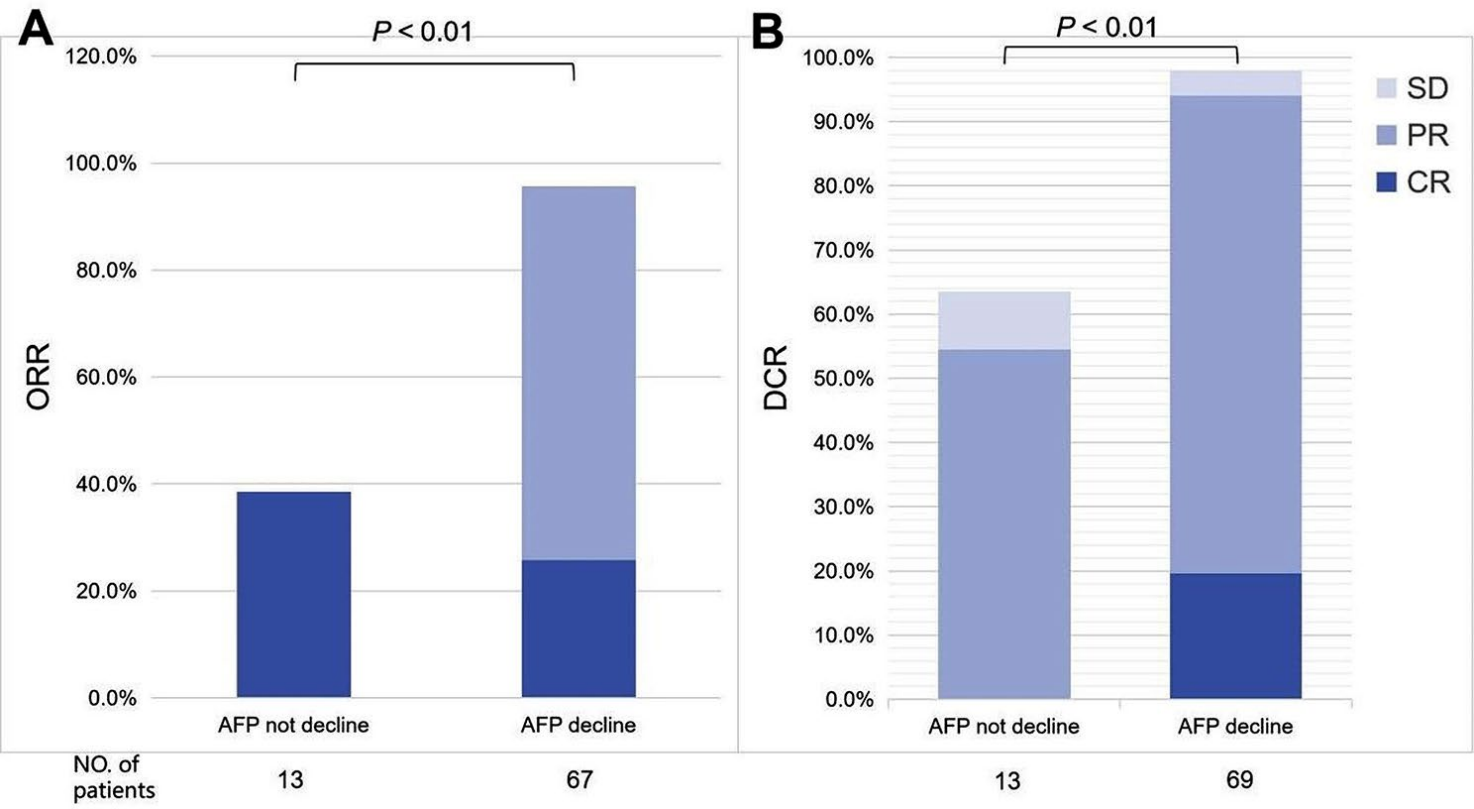
Association between AFP level and imaging response. Patients with reduced AFP levels had significantly higher ORR (**A**) and DCR (**B**). ORR, objective response rate; DCR, disease control rate

Before propensity score matching, the ORR was higher in the TLC group than in the TACE group (88.9% vs. 30.5%, *P* < 0.001). The difference of DCR between the two treatment groups was also statistically significant (95.9%% vs. 69.5%, *P* < 0.001). After propensity score matching, the ORR of TLC group and TACE group were 88.6% and 28.6%, respectively (*P* < 0.001). DCR were 94.3% and 72.9% respectively (*P* = 0.010) (Table 2). Early decline in AFP levels was strongly associated with subsequent imaging response. Patients with reduced AFP levels had significantly higher ORR (95.7% vs. 38.5%, *P* < 0.01) and DCR (98.6% vs. 76.9%, *P* < 0.01) (Fig. 2).

**Table 2:** Summary of best response

Variable	Before PSM				After PSM		
	TLC (*n* = 97)	TACE (*n* = 151)	*P* value		TLC (*n* = 70)	TACE (*n* = 70)	*P* value
Complete response (CR)	22 (22.7%)	9 (6.0%)			15 (21.4%)	4 (5.7%)	
Partial response (PR)	64 (66.0%)	37 (24.5%)			47 (67.2%)	16 (22.9%)	
Stable disease (SD)	7 (7.2%)	59 (39.1%)			4 (5.7%)	31 (44.3%)	
Progressive disease (PD)	4 (4.1%)	46 (30.5%)	< 0.001		4 (5.7%)	19 (27.1%)	< 0.001
Overall response rate (ORR)	86 (88.9%)	46 (30.5%)	< 0.001		62 (88.6%)	20 (28.6%)	< 0.001
Disease control rate (DCR)	93 (95.9%)	105 (69.5%)	< 0.001		51 (94.3%)	51 (72.9%)	< 0.001
Operation rate	43 (44.3%)	-			29 (41.4%)	-	

PSM - propensity score matching

Before propensity score matching, the surgical conversion rate of TLC group was 44.3% (43 / 97) and 41.4% (29 / 70) after propensity score matching. Of the surgery patients, 41.9% (18 / 43) had a postoperative pathology suggesting complete response. Comparison of baseline data between surgical conversion and non-surgical conversion patients, surgical conversion group had significantly fewer large vascular invasions (42.0% vs. 67.5%, *P* = 0.031) and fewer tumor number (31.6% vs. 57.5%, *P* = 0.039). During a median follow-up of 15.3 months after surgery (0.6–29.3 months), 8 patients developed intrahepatic recurrence, of which 1 patient developed bone metastases. The typical cases are shown in Supplementary Fig. 1.

### Safety Assessment

Table 3 shows the frequency of adverse events after the start of treatment in both groups. The most common adverse events in the TLC group at all levels were impaired liver function, such as elevated transaminase (including AST / ALT) (97.9%/85.6%), decreased albumin (62.9%) and elevated total bilirubin (54.6%); The second was post intervention syndrome, such as pain (62.9%), hypertension 27.8%) and fever (16.5%). Other grade 3 or higher AEs were not significantly different. AEs of importance grade ≥ 3 observed were AST increased, ALT increased, total bilirubin increased, hypertension, albumin decreased, pain, and hand-foot syndrome. Most patients’ symptoms disappeared in a short time. Targeted and immune-related adverse events, including hand-foot syndrome and reactive cutaneous capillary endothelial proliferation (RCCEP), were 22.7% and 8.2%, respectively (Table 3).

**Table 3:** Treatment-related adverse events

Adverse events	Any Grade				Grade 3/4		
	TLC(*N* = 97)	TACE(*N* = 151)	*P*-value		TLC(*N* = 97)	TACE(*N* = 151)	*P*-value
Total bilirubin increased	53 (54.6%)	120 (79.5%)	< 0.001		1 (1.0%)	37 (24.5%)	< 0.001
Alanine aminotransferase increased	83 (85.6%)	133 (88.1%)	0.651		27 (27.8%)	68 (45.0%)	0.017
Aspertate aminotransferase increased	95 (97.9%)	143 (94.7%)	0.171		55 (56.7%)	85 (56.3%)	0.215
Albumin decreased	61 (62.9%)	140 (92.7%)	< 0.001		0	1 (0.7%)	0.492
Hypertension	27 (27.8%)	39 (25.8%)	0.936		6 (6.2%)	19 (12.6%)	0.215
Fever	16 (16.5%)	69 (45.7%)	< 0.001		0	0	-
Pain	61 (62.9%)	136 (90.1%)	< 0.001		5 (5.2%)	15 (9.9%)	0.098
Proteinuria	7 (7.2%)	0	< 0.001		0	0	-
Fatigue	16 (16.5%)	33 (21.9%)	0.392		0	0	-
Hand-foot syndrome	22 (22.7%)	0	< 0.001		1 (1.0%)	0	0.320
Skin and subcutaneous tissue diseases	8 (8.2%)	0	< 0.001		0	0	-
Diarrhea	10 (10.3%)	6 (4.0%)	0.037		0	0	-
Nausea	21 (21.6%)	48 (31.8%)	0.156		0	0	-
Decreased appetite	5 (5.2%)	17 (11.3%)	0.182		0	0	-
Weight drop	21 (21.6%)	37(24.5%)	0.234		0	0	-
Edema peripheral	7 (7.2%)	15 (9.9%)	0.725		0	0	-

### Comparison of PFS and OS between the two groups

**Fig. 3:**
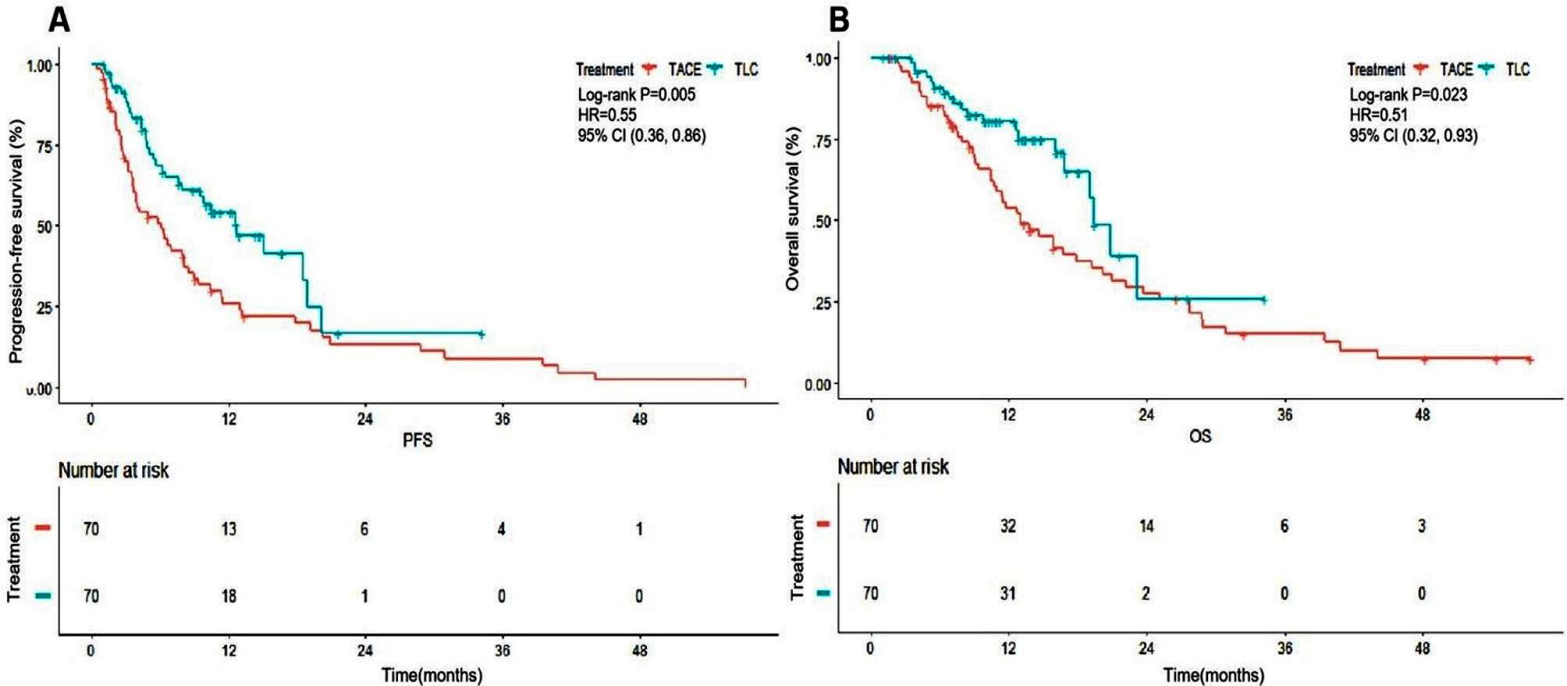
Kaplan Meier analysis in TACE + lenvatinib + camrelizumab group and TACE group (**A**) Progression free survival (PFS) and (**B**) cumulative overall survival (OS)

After matching, the median PFS in the TLC group was longer than that in the TACE group (12.7 vs. 6.1 months, *P* = 0.005) (Fig. 3A). The PFS rates at 6-, 12- and 18- months in the TLC group were 68.5%, 54.1% and 41.2%, respectively, and 50.6%, 25.8% and 19.6% in the TACE group. The median OS in the TLC group was longer than that in the TACE group (19.4 vs. 13.0 months, *P* = 0.023). The 12-,18- and 24-months OS rates of TLC group were 80.3%, 64.9% and 25.9% respectively, and the TACE group were 53.8%, 37.4% and 27.6%, respectively (Fig. 3B).

**Fig. 4:**
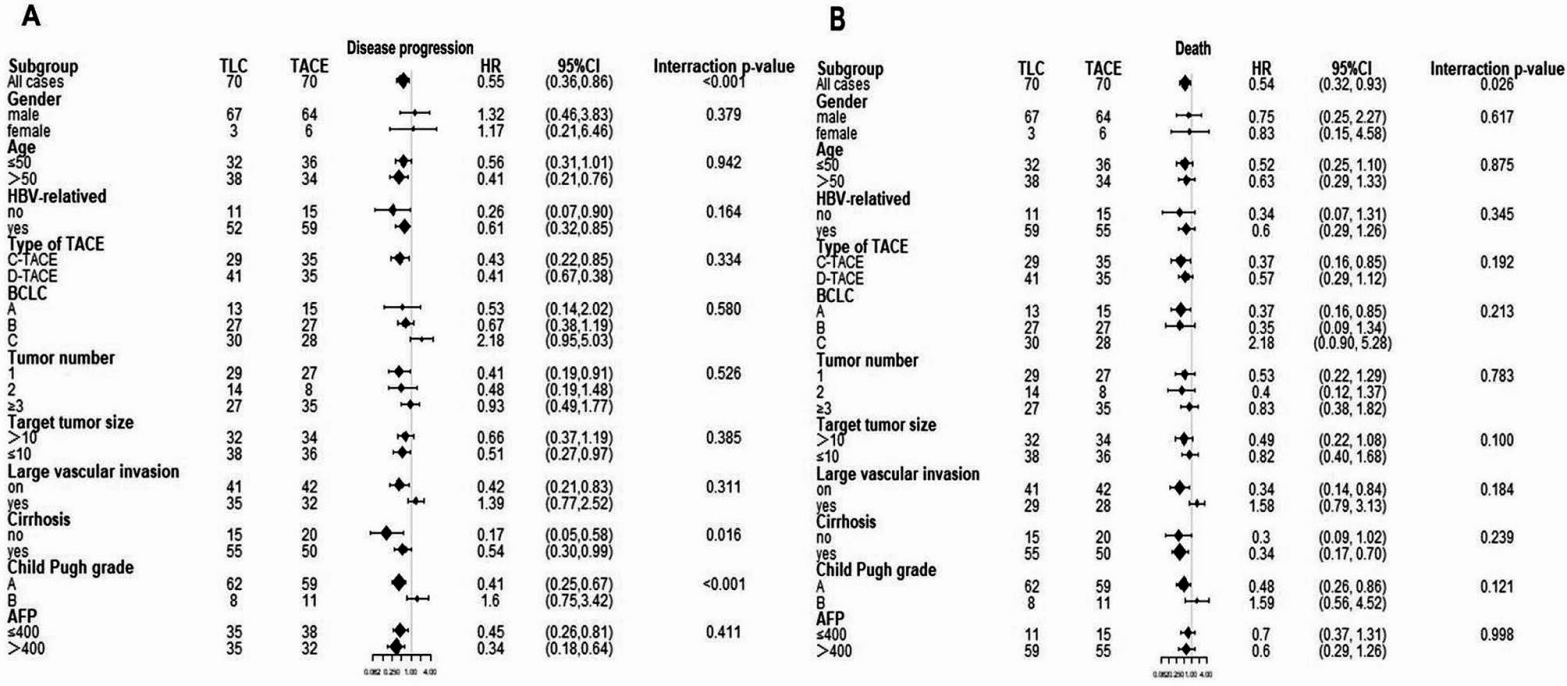
Subgroup analysis in the matched cohort. Subgroup analysis for disease progression (**A**) or death (**B**)

Univariate analysis of PFS in 140 patients showed that large vascular invasion (no / yes), stage of BCLC (A/ B/ C), treatment mode (TACE / TLC) and AFP (> 400 / ≤400ng/ml) were relevant factors (Fig. 4A). All of the subgroups favored TCL group. When these variables were included in the multivariate analysis, the results showed that treatment modality (TACE/TLC) (HR = 0.42, 95% CI: 0.28–0.62, *P* < 0.001) was an independent factor for PFS. Univariate analysis of OS showed that large vascular invasion (no / yes), stage of BCLC (A/ B/ C), (TACE / TLC) and AFP (> 400 / ≤400ng/ml) were OS related factors (Fig. 4B). After multivariate analysis, the results showed that treatment mode (TACE / TLC) (HR = 0.32, 95% CI = 0.20–0.51, *P* < 0.001) was an independent influencing factor of OS (Table 4).

**Table 4:** Multivariable COX model based on treatment options for progression-free survival and overall survival

Outcome	Non-adjusted		Adjust I		Adjust II	
	HR (95% CI)	*P*-value	HR (95% CI)	*P*-value	HR (95% CI)	*P*-value
progression-free survival						
TACE	1		1		1	
TLC	0.44 (0.31, 0.63)	< 0.001	0.42 (0.28, 0.62)	< 0.001	0.39 (0.26, 0.57)	< 0.001
overall survival						
TACE	1		1		1	
TLC	0.41 (0.26, 0.63)	< 0.001	0.32 (0.20, 0.51)	< 0.001	0.40 (0.24, 0.65)	< 0.001

HBV: Hepatitis B virus; BCLC: Barcelona Clinic Liver Cancer; TBIL: Total bilirubin; ALB: Albumin; PT: Prothrombin time; AFP:α-fetoprotein

Stratified analyses according to patient characteristics are shown in Fig. 4. Effects were similar across most subgroups; however, the PFS in TLC group appeared to be more pronounced among patients who did not have cirrosis than among patients who had cirrosis (*P* = 0.016 for the interaction) (Fig. 4A).

## Discussion

Recent advancements in locoregional therapy, targeted therapy, and immunotherapy have opened new avenues in treating uHCC. Combination therapies such as local therapy combined with targeted therapy and targeted therapy combined with immunotherapy have demonstrated positive outcomes in uHCC [24–29].

In this study we revealed that according to mRECIST, ORR (88.6% vs. 28.6%, *P* < 0.001) and DCR (94.3%% vs. 72.9%, *P* < 0.001) in TLC group were significantly higher than in TACE group after PSM. TLC was helpful to prolong PFS (12.7 vs. 6.1 months, *P* = 0.005) and OS (19.4 vs. 13.0 months, *P* = 0.023) in uHCC, and the treatment regimen is an independent predictor of improving PFS and OS. PFS in TLC group appeared to be more pronounced among patients who did not have cirrosis than among patients who had cirrosis (*P* = 0.016 for the interaction). In addition, TACE combined with lenvatinib and camrelizumab did not produce unexpected TREA, which was similar to the use of these treatments alone or in combination. The incidence of hand foot syndrome and RCCEP were 22.7% and 8.2%, which was not higher than that in previous studies [24–30]. The reason why the incidence of any grade of TRAE and ≥ grade 3 TRAE is higher than that of the above methods may be the post embolism syndrome caused by TACE, which is relieved in a short time after symptomatic treatment. It shows that TACE combined with lenvatinib and camrelizumab are safe.

In recent years, conversion therapy has garnered interest in the management of uHCC. Some studies have shown that the 5 years OS rate of surgical resection of converted HCC is not significantly different from that of patients with initially resectable HCC [31].At present, the surgical convertion rate of different triple therapies ranges from 25.7 − 50% [24–26, 28, 29, 32].The results of this study showed that the surgical conversion rate was 44.3%, which was consistent with the above studies. Among them, 41.9% (18 / 43) of successfully transformed patients achieved complete pathological remission.

Currently, there is no exact research report on the mechanism of TACE in combination with lenvatinib and camrelizumab. The potential mechanisms may be as follows: TACE and TKI (lenvatinib) have complementary effects, on the one hand, TACE treatment can cause tumor ischemia, hypoxia and necrosis, and the environment of ischemia and hypoxia will increase the level of hypoxia inducible factor-1 (HIF-1), which may increase synthesis of vascular endothelial growth factor and platelet-derived growth factor, leading to the regeneration and progression of tumor microvessels [33, 34]. However, levatinib contribute to inhibit signal transduction pathways such as VEGF and FGF and play an anti-angiogenesis and direct anti-tumor role [35]. At the same time, TACE + PD-1 inhibitor has a synergistic effect, which increased the expression of PD-1 and PD-L1 in tumor cells. Binding of PD-1 to PD-L1 leads to immune escape and promotes tumor growth [36, 37]. Carrazumab suppresses tumor cell immune escape by blocking PD-1 binding to PD-L1, thus enhancing the tumor immune responses [38] .

To date, there is no reliable and effective biomarker for predicting the efficacy of PD-1 inhibitors in HCC. Interestingly, in our study, the decrease in AFP levels following the second course of TACE therapy was found to be an early and effective predictor of treatment outcome. It may suggest that PD-1 inhibitors exerts an antitumor effect at early stage.

According to the analysis of patients’ clinicopathologic characteristics undergoing transformation treatment and surgery, AFP increased (> 8.78ng / ml) in 83 patients before treatment, and decreased to normal in 40 patients (48.2%) after treatment, which is higher than the proportion of 35.9% in previous studies [39]. Previous studies showed that the tumor diameter was 5.1–6.5 cm, and the incidence of MVI was 55% [40]. In patients undergoing hepatectomy with MVI, the 5 years OS rate and RFS rate were significantly lower than those without MVI (OS rate: 38.4% vs. 66.1%, *P* < 0.0001; RFS rate: 15.8% vs. 28.6%, *P* < 0.0001) [41]. Most of the patients in this study had tumor diameters > 5 cm, and the incidence of MVI was 9.3% (4 / 43) in postoperative pathology, which was MVI type M1. For patients with portal vein tumor thrombus, according to the different types of tumor thrombus (Japanese liver cancer association classification VP1-4), the median RFS was 0.38–1.23 years, the median OS was 1.44–2.87 years, and the OS rates of 1, 3 and 5 years were 61.3 − 74.8%, 35.2 − 49.1% and 25.6 − 39.1% respectively [42]. In the group with BCLC 0/A, recurrence-free survival (RFS) at 1, 3, and 5 years was 74%, 43%, and 31%, respectively, and OS rate at 1, 3, and 5 years was 89%, 70%, and 52%, respectively [43]. In this study, preoperative imaging showed that 2 patients had different degree of hepatic vein tumor thrombosis (HVTT) regression and reduction of scope, 8/22 patients had demotion of portal vein tumor thrombosis (PVTT) from type III to type II, and 16/22 patients had different degree of PVTT regression and reduction of scope. Postoperative pathological results showed complete necrosis of PVTT in 8 patients. This suggests that these high-risk recurrence factors may be transformed into non-high-risk factors after TACE combined with lenvatinib and camrelizumab, so as to improve the curative effect and improve the prognosis of patients, but further research is needed to confirm.

Our database included patients from China, who were treated in different settings. The range of patients was broad: men and women, from teenagers to elderly people were included, and the major exposure categories were well represented. The stage of BCLC at baseline ranged from A to C, and liver function status from child-pugh grade A to B.

There are also some limitations to this study. First, retrospective studies may have selected biases; Second, this is a single-center, small and retrospective study, which needs to be further verified by large, multi-center and prospective studies; Third, the follow-up time is relatively short, and the long-term effect needs to be further studied.

## Conclusion

In conclusion, according to our results, the combined treatment of TACE, lenvatinib and camrelizumab is safe and effective for uHCC and may be a potentially effective transformation treatment.

### Electronic supplementary material

Below is the link to the electronic supplementary material.

Supplementary Material 1

## Data Availability

The data are not publicly available because it contains information that could compromise the privacy of study participants.
