# Supplementary material for: TACE combined Lenvatinib plus Camrelizumab versus TACE alone in efficacy and safety for unresectable hepatocellular carcinoma: a propensity score-matching study
Source: BMC Cancer. 2024 Jun 11;24:717. doi: 10.1186/s12885-024-12484-3 (PMC11165855; doi:10.1186/s12885-024-12484-3)
Supplement: Supplementary file 1 — Supplementary Material 1 [file 12885_2024_12484_MOESM1_ESM.docx]

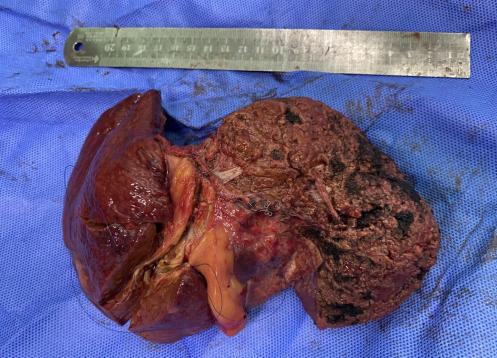

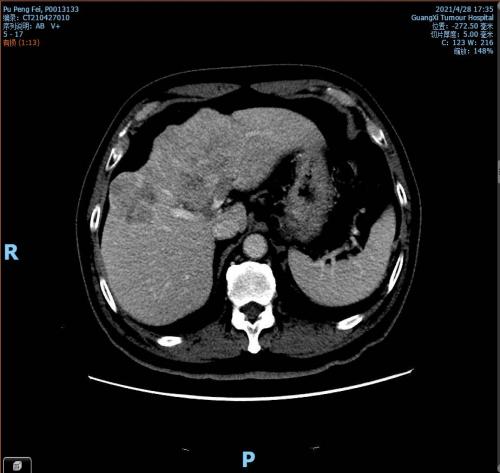

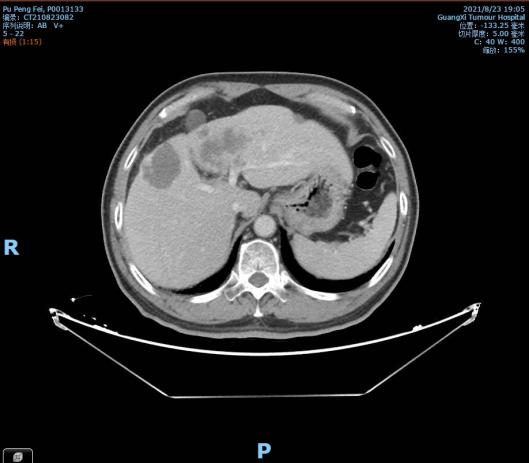

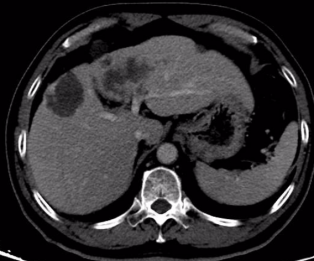

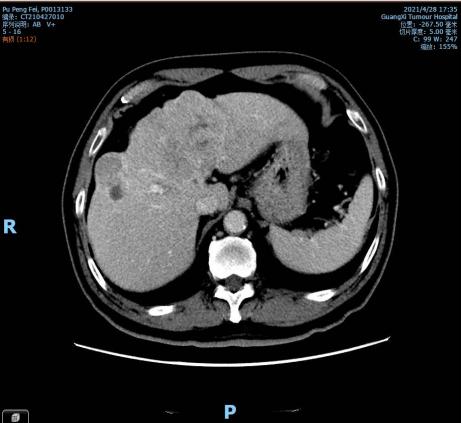

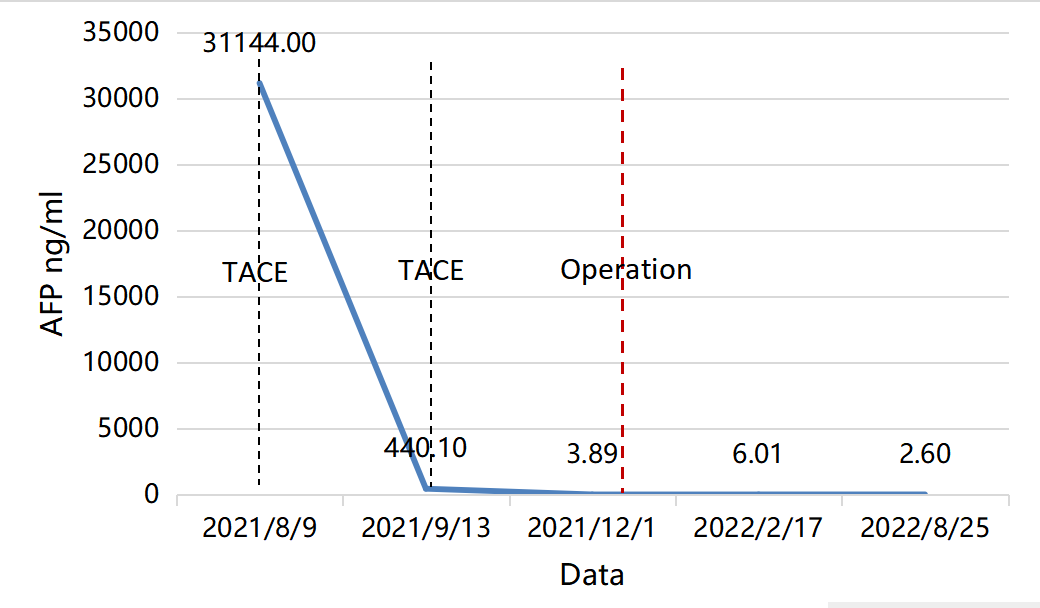

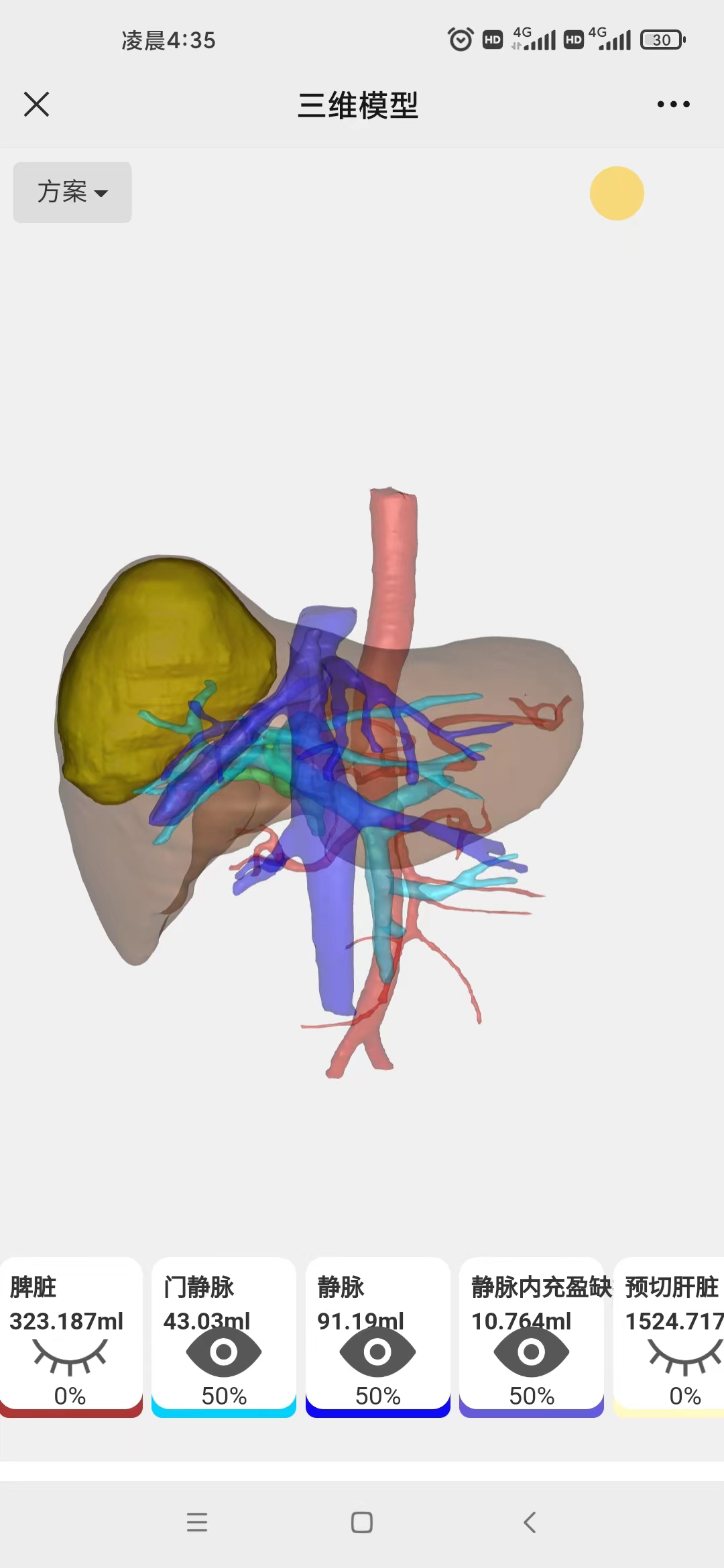

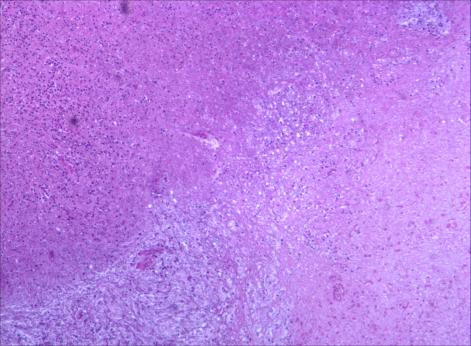

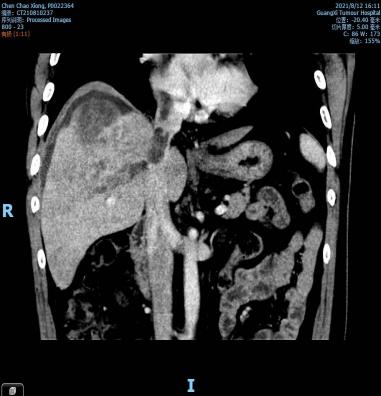

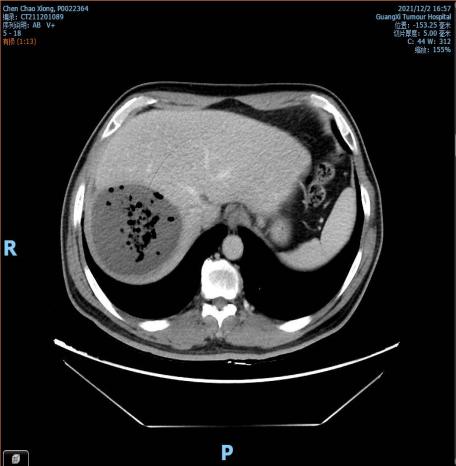

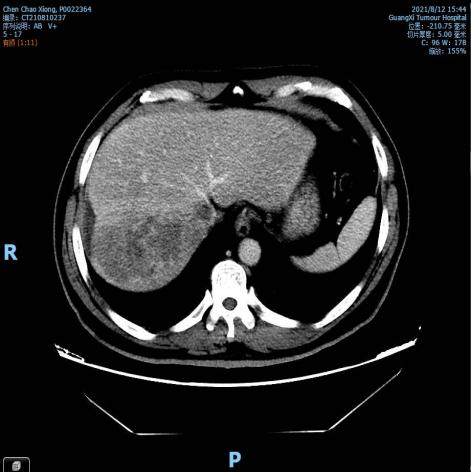

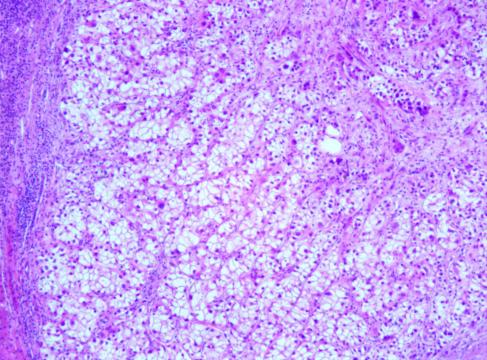

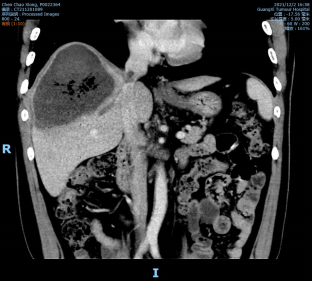


Change of AFP levels

**A**

Pretreatment CT

3D reconstruction

H&E staining of resected specimen

**Supplemental Figure 1**. Two representative cases. **(A)** The patient was diagnosed with HCC of the right hepatic lobe, invading the hepatic vein and the inferior vena cava (yellow arrow pointing IVCTT). After conversion therapy for 3.5 months, the primary tumors showed no arterial enhancement on contrast-enhanced CT, and the IVCTT regressed. Right hemihepatectomy was performed. H&E staining of the surgically resected specimen showed tumor and IVCTT complete coagulative necrosis (pathologic complete response). **(B)** The patient was diagnosed with multiple hepatocellular carcinoma involving the left lobe and invading the left branch of the portal vein (red arrow pointing PVTT). After conversion therapy for 4.0 months, the portal vein tumor thrombosis regressed and tumors shrinked. Extended left hemihepatectomy was performed.H&E staining of the surgically resected specimen showed hepatocellular carcinoma. PVTT is completely necrotic ,viable tumor cell rate 2.5% (major pathologic response).

IVCTT: inferior vena cava tumor thrombus, PVTT:portal vein tumor thrombus,

**B**

Resected specimen

H&E staining of resected specimen

4.0 months (Before surgery)

Pretreatment CT

3.5 months (Before surgery)
